# Exploring the Feasibility and Acceptability of Technological Interventions to Prevent Adolescents’ Exposure to Online Pornography: Qualitative Research

**DOI:** 10.2196/58684

**Published:** 2024-11-05

**Authors:** Jake Turvey, Dana McKay, Sarah T Kaur, Natasha Castree, Shanton Chang, Megan S C Lim

**Affiliations:** 1 Burnet Institute Melbourne Australia; 2 Royal Melbourne Institute of Technology Melbourne Australia; 3 Portable Collingwood Australia; 4 Monash University Melbourne Australia; 5 University of Melbourne Parkville Australia

**Keywords:** pornography, sexual health, young people, co-design, online safety, age verification, adolescents, attitudes, acceptability, usability, feasibility

## Abstract

**Background:**

Amid growing concern over children’s access to online pornography, policy makers are looking toward new and emerging technological concepts for unexplored solutions including artificial intelligence and facial recognition.

**Objective:**

This study sought to explore and ideate emerging technological interventions that are feasible, acceptable, and effective in preventing and controlling the exposure of young people to online pornographic material.

**Methods:**

We conducted a series of qualitative co-design workshops with both adult (n=8; aged 32-53 years) and adolescent participants (n=4; aged 15-17 years) to ideate potential technological interventions that are feasible, acceptable, and effective at preventing and controlling the exposure of young people to online pornographic material. A story stem methodology was used to explore participants’ attitudes toward two unique technological prototypes.

**Results:**

Participants expressed a generally favorable view of the proposed technological concepts but remained unconvinced of their overall utility and effectiveness in preventing the intentional viewing of pornography by young people. Age-appropriate parent-child conversations remained participants’ preferred approach to mitigating potential harms from pornographic material, with parents also expressing a desire for more educational resources to help them better navigate these discussions. User privacy and data security were a primary concern for participants, particularly surrounding the use and collection of biometric data.

**Conclusions:**

Internationally, policy makers are taking action to use age assurance technologies to prevent children’s access to online pornography. It is important to consider the needs and opinions of parents and young people in the use and implementation of these technologies. Participants in this study were generally supportive of new and emerging technologies as useful tools in preventing the accidental exposure of young people to online pornographic material. However, participants remained less convinced of their ability to avert intentional viewing, with substantial concerns regarding technological efficacy, adaptability, and user privacy. Further, co-design and prototype refinement are needed to better understand user acceptability and comfortability of these new technological interventions, alongside additional research exploring sociocultural differences in information needs and user experiences.

## Introduction

### Background

Internationally, the median age of first intentional viewing of pornography is around 13 years; however, the majority of young people are exposed unintentionally at an even younger age [1-5]. Accidental viewing may be more harmful than intentional access due to the shock and distress experienced upon unexpected or unwanted exposure to pornographic material [6]. Consultations by the Australian eSafety Commissioner found that it was widely considered that pornography is harmful for younger children, but that the balance between harm and benefit is more nuanced for older teens and adults [3].

In recent years, the use of restrictive technology, such as internet filters, has become a common approach to preventing children’s early exposure to pornography [7]. However, these technologies are very unpopular with young people and their parents. A 2021 study, for example, found that only 22% of young Australians supported internet filters blocking pornographic content at a national level, while a 2014 study from the United States found that two-thirds of adults opposed censorship of pornography [8,9]. In research with parents in Australia, the United Kingdom, and the United States, very few had used internet filters [10-12]. A key barrier to use was the belief that the technology was ineffective and could be easily bypassed by young people, with other concerns including the impact on the parent-child relationship, data security and privacy, and a lack of perceived need [2,3,10-12].

But technology continues to evolve, with advances, including natural language processing and visual processing, and products, like age verification and kid-safe internet searches, gaining increased attention [13-17]. Technologies can also incorporate behavioral interventions, such as pornography literacy education or self-monitoring and metrification [18,19]. These technologies offer unexplored opportunities and barriers to families in keeping children safe. Furthermore, there have been renewed efforts from governments to implement policy solutions. For example, age verification for access to online pornography is legislated in Germany and France [20,21]. In 2023, the Australian eSafety Commissioner published the Age Verification Roadmap, which considered the feasibility of and evidence for mandatory age verification mechanisms for access to online pornographic content throughout Australia [3]. In 2024, the Australian Government announced an age assurance trial to explore technologies to prevent children’s access to online pornography [22]. With these policies being implemented, it is vital to consult the community about how they could be applied. It is within this context, that our paper contributes to an emerging evidence base, and provides important insights into the acceptability and feasibility of new-age verification technologies.

### Research Aims

This study seeks to gain a better understanding of user attitudes to emerging technology and policies relating to pornographic access and identify barriers and opportunities for implementing interventions. We used co-design methods to work with parents and young people to explore how these technologies could be used. This research project aimed to ideate technological interventions that are feasible, acceptable, and effective in preventing and controlling the exposure of young people to pornography.

## Methods

### Phase 1: Discovery and Concept Design

In the first phase of this study, the research team sought to establish what current and emerging technological solutions have been proven or have shown promising efficacy toward the prevention of young people from online sexual harm and exposure to online explicit material. Researchers from the Monash Sustainable Development Institute Evidence Review Service were engaged in July 2022 to conduct a rapid systematic review of contemporary literature and practice, with additional information collected via expert interviews and bibliographic analysis [23]. A final report was delivered to the research team in November 2022.

Following the discovery process, the research team evaluated the existing technological solutions, as well as emerging developments, contexts, and attitudes identified through the evidence review to create a series of speculative prototypes to be tested with research participants. Two of these technological concepts were developed and refined (Textbox 1). The purpose of these concepts is not to present the best possible prototypes but to generate critical discussion about different features of technology.

**Technological concepts.**

**“SafeAgeID”**

This concept seeks to harness facial imagery processing techniques to analyze a user’s facial profile and determine an approximate age. This may include measuring ratios between facial features, detecting the degree of wrinkling on a user’s face, or measuring a range of points around the nose and eyes. It is proposed that, when installed on users’ devices, the “SafeAgeID” app would require a facial scan validation prior to accessing explicit content. If a young person attempts to view restricted materials and fails a facial validation assessment, a notification will be sent to the young person’s parent or guardian with information about the attempted access, and the material will be blocked from view on the young person’s device.

**“PornScreenPro”**

This concept seeks to combine both parental monitoring software and internet filters into a single app. The “PornScreenPro” technology would allow parents or guardians to set certain restrictions and limits on the type of material available to view on each device and would also allow the active or passive monitoring of explicit content access, generating regular reports and notifications about trends and viewing activity. This technology could also be used by adults as a form of self-monitoring and reflection, similar to other forms of self-metrication, for example, step counting [18].

### Phase 2: Co-Design Workshops

The second phase of the study involved generative co-design workshops. Data from these workshops was used to generate two outputs: (1) a thematic exploration of participant attitudes toward new technological interventions (described in this paper), and (2) a set of design principles and specifications for further development of potential low-fidelity technological prototypes (not described in this paper).

### Recruitment

Participant recruitment was facilitated by an external research recruitment agency based in the Melbourne area. The agency sent a brief advertisement to an existing network of suitable participants, and those interested in participating were then directed to complete an online expression of interest survey through the REDCap (Research Electronic Data Capture; Vanderbilt University) platform. Respondents were eligible to participate if they were either adolescents aged between 13 and 18 years or a parent or legal guardian of an adolescent aged between 8 and 16 years. Participants were required to be proficient in English and experiencing economic hardship or social disadvantage, determined through self-reported access to a government-issued health care card or receipt of income support at the time of research. Socioeconomic disadvantage was included because of evidence that digital health interventions can exacerbate inequity when the “digital divide” is not addressed [24,25]. Therefore, we included those experiencing disadvantage to purposefully co-design interventions that met this group’s needs.

Research recruiters contacted suitable candidates following their expression of interest to confirm their eligibility and availability and to provide additional information about the workshop content and structure. Confirmed participants were then sent an email invitation with selected workshop times, along with participant information and consent forms. All participants provided informed written consent including informed parental consent for all participants younger than 18 years of age.

### Participants

In total, 12 individuals took part in the research workshops: 8 parents and 4 adolescents. Parents were aged between 32 and 53 years, 75% (n=6) of parents identified as female, 50% (n=4) of parents had a tertiary education, and 38% (n=3) of parents spoke a language other than English at home. Adolescent participants were all aged between 15 and 17 years, and 75% (n=3) of adolescents identified as female. All were current high school students, with 25% (n=1) of adolescents speaking a language other than English at home.

### Workshop Procedure

In mid-2023; two 90-minute workshops were held online via Zoom (Zoom Video Communications, Inc) with participating parents across consecutive weeks. A third in-person workshop was held over a 2-hour period with adolescent participants at an inner-city university campus.

Each workshop was facilitated by a multidisciplinary team including a public health researcher, a digital information researcher, and a design practitioner. Across all workshops, researchers used a story-stem completion methodology to explore participants’ perspectives toward the proposed technological concepts [26]. This relatively novel qualitative method presents participants with an incomplete hypothetical scenario and invites participants to complete the narrative by drawing on their own unique experiences, expectations, and mean-making processes [27]. This methodology was specifically selected by the research team as it not only allowed insight into the perceived acceptability and feasibility of the two technological concepts but also generated a greater understanding of each participant’s broader attitudes toward young people’s online interactions and themes of adolescent sexual development. As a speculative method, story-stem completion is particularly useful for exploring concepts or technologies that may not exist or that participants may not have experienced before [28]. As an approach that uses a third-person narrative, it is also excellent for eliciting participants’ knowledge, thoughts, and feelings about sensitive themes [29].

All participants were first given a brief overview of the technological concepts being evaluated including their proposed use and expected functions. A series of story stems were then presented one-by-one to participants, featuring two hypothetical, yet realistic scenarios (Textbox 2). The names in the story stems were deliberately chosen for their gender-neutral associations in Australian English, allowing participants to form their views and apply their gendered lenses to the story stems. Facilitators read aloud the first scenario (part A), and participants were then encouraged to continue the hypothetical story arc individually in a separate worksheet, drawing upon their own experiences and perspectives to complete the narrative. Each anonymous response was collected from participants, before being read aloud by the facilitators with group discussions and reflections to follow. In poststory discussions, facilitators prompted participants to consider and discuss the likelihood of the stories happening in real life, preferred outcomes of stories, how their responses would differ in different circumstances (eg, older or younger child), how the stories made them feel, modifications they would make to the concepts, and other unintended consequences of these technologies. Participants were then given a second related story stem (part B) that continued the first story arc to elicit further insights following slight changes in the hypothetical narrative. Once again, participant responses were collected and discussed as a group, with researchers prompting deeper discussions as needed. This process was repeated for the second technological concept, with new hypothetical scenarios used. Both parents and adolescent participants responded to the same story stem scenarios, with parents completing one concept per workshop, while adolescents completed all story stems in a single workshop.

Following the conclusion of each workshop, the research facilitators also participated in a collective debrief lasting between 20 and 40 minutes, in which key discussion themes and participant responses were further explored. This provided the opportunity for the research team to not only share their professional perspectives on the key insights gained from each workshop but also reflect on the workshop process and refine any minor issues for future sessions.

**Workshop story stems.**

**Technological concept 1: “SafeAgeID” facial scan recognition**

- Part A
  Harper is 8 years old. At lunchtime, Harper heard the term “threesome” being used by friends but did not understand what it meant. At home, Harper uses the family iPad and types “threesome” into Google.
  Harper finds a video in the search results and clicks on the link. The iPad suddenly blurs everything on the screen and asks for a facial scan before being allowed to continue to view the explicit video.
- Part B
  The age verification technology scans Harper’s face and decides Harper is probably younger than 18 years and blocks the porn site. The SafeAgeID app instructs Harper to ask for an adult’s help to proceed.
  SafeAgeID app sends a notification to Harper’s parents about an attempted porn site viewing, with the date, time, porn video’s URL, and the image of Harper’s face from the scan.

**Technological concept 2: “PornScreenPro” monitoring platform**

- Part A
  Jo is 13 years old and uses the family computer to watch videos and games, and do schoolwork. Jo clicks on a link friends have shared for a porn video. A parenting app called “PornScreenPro” is installed on the computer, and it sends Jo’s parents a notification that porn content has been detected as part of their child’s internet activity.
  Along with the notification message, PornScreenPro sends guidance to Jo’s parents on how to talk to a 13-year-old about pornography and internet safety in an age-appropriate way.
- Part B
  Jo is now 19 and watches porn fairly regularly. Lately, Jo has been watching more porn and finding that the content needs to be more graphic or extreme in order to “get off.”
  Jo’s phone has a default activity monitor for activities like running and walking, time spent on social media, TV shows, movies, and porn. Jo gets a notification message, “You have watched porn for 36 hours this month, that is 22 hours more than last month.”
  Jo can look at the advice the app offers on reducing pornography use. It also has information about porn’s impact on relationships and expectations around sex. It can also suggest pornography that is less extreme or violent.

### Data Collection and Analysis

All data, including debrief discussions, workshop audio recordings, and story stem responses, were transcribed and checked for accuracy by researchers. A collaborative iterative thematic analysis approach was adopted to capitalize on the highly multidisciplinary perspectives of the research team [30]. This analysis process was undertaken by two researchers (JT and STK) from the public health and design and technology fields, respectively, with the aim of better integrating specific technological design implications alongside broader themes of young people’s sexual health and development.

Following transcription, researchers first familiarized themselves with the data before creating an independent set of initial codes using NVivo 14 software (Lumivero) and the Miro online platform. The research team then met to discuss this preliminary analysis and to compare initial codes and emerging themes. A second, more extensive round of independent thematic analysis was then undertaken, with initial codes refined and developed into more distinct themes. The research team once again met to discuss the corresponding analyses, and following minor revisions, merged the datasets into a single thematic framework that is reflective of the collective perspectives and understandings of the research team [31].

### Ethical Considerations

Ethics approval was granted by the Alfred Health Human Research Ethics Committee (ID 663/22). All participants gave informed consent. All adult participants were provided with an AU $100 (US $68) gift voucher as an appreciation for their time, while adolescent participants were provided with an AU $120 (US $81) gift card for their contributions and to compensate for the additional travel expenses. All data were deidentified.

## Results

### Overview

Three key themes were identified by researchers from the data, with several subthemes further explored. The key themes and subthemes are outlined in Textbox 3 below.

**Key themes and subthemes.**

**Theme 1: limitations of technology**

- Workarounds and circumvention
- Perceived utility of self-monitoring technology

**Theme 2:** **technology as a secondary tool**

- First line of defense against accidental exposure
- Age-appropriate conversations remain paramount
- Additional support for parents and guardians

**Theme 3: customizable and secure design**

- Privacy and data security
- Configurable options for a diversity of users

### Theme 1: Limitations of Technology

#### Workarounds and Circumvention

Throughout each workshop, participants readily identified limitations that the proposed technologies may encounter when applied to real-world conditions. Participants saw the clear potential for these technologies to prevent accidental exposure. However, most mentioned the inability of these technologies to prevent the intentional access of pornography and other online explicit content. Both adult and adolescent participants strongly believed that young people were likely to easily circumvent the restrictive barriers of the proposed technologies, especially when deliberately seeking to view explicit content.

It’d be pretty easy to get around the face IDs if you really wanted to see something.

Adolescent

The workarounds kind of rang true to me, that [young people] would try to get around the technology in some way.

Parent

Participants described the various ways in which young people were likely to bypass both technological concepts including gaining access through older or alternative devices, seeking access through older siblings or friends, or convincing parents to temporarily disable the technology. Further strategies to navigate the restrictive technologies included using alternative search terminologies such as slang words, emojis, or languages other than English.

...the other thing I did note that was interesting was how the child could then try another way of searching for the [explicit] word … for young teenage boys, an eggplant [emoji] signifies something different so, you know, it’s the different connotations that people may use, and these technologies will not, or cannot really prevent.

Parent

Similarly, several participants also questioned the ability of both technological concepts to prevent harmful exposure through different mediums such as adult cartoons or anime, text-based content, and audio recordings. There was also concern that other forms of harmful online activities, such as sexting or acts of child grooming, would go uncensored, as they were not as easily identifiable as traditional image-based pornographic content.

I feel like we’re a bit reliant on this technology and only the imagery, but not the text sort of content.

Parent

...you think you’re providing this safe platform, whereas they could still go and look up a whole written story about what a threesome was, and that wouldn’t be blocked.

Parent

#### Perceived Utility of Self-Monitoring Technology

When discussing the merits of the “PornScreenPro” concept, both adult and adolescent participants questioned the utility of such monitoring technologies, particularly when applied to older adolescents with a developing sexual identity and curiosity for pornographic content.

I think the whole concept of the [PornScreenPro] app to a teenager or young adult is not something that is suitable at that age, especially if it’s a male. They’ve just finished high school, the world is their oyster, they’re exploring, you know, their sexuality, meeting up with friends, learning different things, and I think the last thing they need or would pay heed to would be an app that sort of prevents them from doing that.

Parent

Participants also felt that older adolescents were unlikely to pause and reflect on their own behaviors and pornographic consumption at such an age, even when notified or provided with supportive resources. Consequently, many felt that the “PornScreenPro” app would quickly become a nuisance to users, leading to the technology either being ignored or disabled with enough time.

One thing that jumped out at me as being really consistent is that the majority of the [story stem] narratives talked about disregarding the [PornScreenPro] app. Young people didn’t care about info on the app, turned off notifications, found them annoying.

Parent

I think if you’re at that stage [of life] you’re not going to see a notification about [harmful pornographic consumption] and be like “ah maybe I’ll look at that,” you’ll just be like “I don’t really care.”

Adolescent

### Theme 2: Technology as a Secondary Tool

#### First Line of Defense Against Accidental Exposure

Despite the limitations identified above, there was still an overarching agreement among both parent and adolescent participants that these novel technological interventions had a place in protecting young people from online harms, with general support for their further refinement and development. Participants discussed how these new technologies could still be beneficial tools, acting as a “first line of defense” against the accidental exposure of younger children to pornography and other explicit content.

Whether the child goes looking for answers when they’re not ready, well hopefully this technology, once its released, it would screen out and block those because they’re not of age yet, or they’re not, you know, ready for it.

Parent

#### Age-Appropriate Conversations Remain Paramount

However, participants reiterated that these apps should not be seen as a “fail-safe” solution and that there was still an important need for close parental oversight and engagement. More specifically, both adult and adolescent participants overwhelmingly agreed that age-appropriate conversations remained the most suitable and preferred course of action for addressing concerns of online harm to young people.

I feel like there’s a danger that people would think that it was a fail-safe technology … and that the most important aspect is that it shouldn’t replace those ongoing conversations about being safe online.

Parent

[Jo’s] parents get the link and are not sure what to do so they approach the situation carefully. They realise that it was an accidental mistake and so are cautious on talking to her but do make it a point in the future to educate her about porn in a safe way. Over the next few days its forgotten and Jo is careful not to click on random links again.

Parent story stem response

Indeed, many of the participant’s hypothetical story-stem responses described an “ideal” outcome whereby parents avoided emotional responses and instead opted for a more empathetic and educational conversation.

[The parents] are glad that this technology has blocked their underage child’s access. Next few days, the parents sit the child down to ask why she went searching and what would she like to know. Parents propose the next time the child is curious, she should approach the parents first.

Parent story stem response

Ideally, after some confusion and perhaps further failed attempts at accessing the content, Harper would feel comfortable enough to ask one of her parents about the definition of the term [threesome].

Adolescent story stem response

Some parents further suggested that adopting these technological apps in their homes would also act as a catalyst for them to have the necessary conversations about online safety with their children before any exposures occur.

...if this technology actually existed you would of kind of have to have some of those conversations already about these filters, like “these things exist, there’s this app on the iPad that will help stop you from seeing inappropriate content.”

Parent

#### Additional Support for Parents and Guardians

Yet, while participants placed significant importance on having these open and informed conversations with young people, several parents also expressed a lack of confidence in their own ability to do so. Many parents felt unprepared for what they described as “difficult” and “awkward” conversations and reflected that they felt less technologically literate in today’s online world.

We [parents] have sort of I guess been brought up with a limited sort of technology and our children are obviously, it’s a second language for them, you know, they understand sometimes more than we do so I feel at times they may be able to actually get around it more than we actually can.

Parent

Parents expressed a desire for more educational resources on how to discuss pornography and internet safety in age-appropriate ways and felt that this information must accompany any internet monitoring technology.

We understand our children but don’t get a good sense of how to educate them in the proper way. They may not really listen.

Parent

Several parents also stated that these conversations remain a shared responsibility, with the school system and educators also needing to play a strong role in delivering appropriate cybersecurity and sexual health education.

Teachers still play critical role within the school to educate the children, the students, rather than the parent. Parents can play a supporting role.

Parent

### Theme 3: Customizable and Secure Design

#### Privacy and Data Security

User safety and data security were a paramount concern for many participants. Several parents expressed a lack of confidence in the ability of third-party apps to protect user information, with numerous participants referencing recent large-scale cyberattacks on Australian organizations, and how these data breaches had further heightened their anxiety regarding online privacy.

So many sites have been hacked lately that we would normally think are really secure, like telecommunications and banking.

Parent

There was a particularly strong apprehension from parents toward the safe storage of biometric data, with facial scans and images of young people seen as especially sensitive. Some felt that the collection and storage of such sensitive biometric data would in fact attract greater attention from cyber-criminals, with the technology acting as a “beacon” for malicious actors to gain access to young users and their valuable personal data.

Having those photos of the children, are they going to be safe? That was my concern as well, just like how everyone mentioned, and too much information being out there so those were my two concerns that I’ve just been pondering about … in some way this technology would be a bit of a beacon for groomers and people wanting to access children.

Parent

While these privacy concerns did not undermine participants’ overall assessment of the utility of the technological interventions per se, participants did articulate a strong desire for further information on the safety mechanisms of the proposed technologies. Many felt that these concerns would need to be thoroughly addressed before they felt comfortable using the different apps.

I think mostly parents would be accepting of it, I think maybe, I think you’d hit the majority of parents, at least yeah, but I still think the biggest concern would be the face ID technology and the collection of data and identity and I think that yeah that would be the biggest concern.

Parent

#### Configurable Options for a Diversity of Users

Additionally, while participants were quite forthcoming with their own thoughts and reflections on the proposed technologies, many were also acutely aware of and frequently vocalized the need to consider alternative perspectives and value systems in the development process. Both parents and adolescents sought to highlight that each person, family, and sociocultural group may hold different attitudes toward sex, pornographic content, and young people’s sexual development more generally. Consequently, participants expressed a desire to see flexible and highly configurable options available within the proposed technologies to suit the individual needs of users and the unique sociocultural requirements of different family groups.

Yeah, because for different households, maybe we say ok I’ve got a 10-year-old, I think my 10-year-old is not ready for that but if another 10-year-old in another household goes “mum and dad, I want to learn about this” then they do the age verification and then its ok for them.

Parent

I hope that if this kind of technology comes out it would kind of help with acknowledging the fact people choose to [use the technology] for different reasons … it depends on why they’re using it, and how they’ve been brought up. I feel that a technology that addresses that kind of diverse range of people using it would be the most impactful like positively.

Parent

## Discussion

### Principal Results

Both adult and adolescent participants in this study expressed a general level of support for the use of age verification technologies in the regulation of pornography and other online materials for young people. This finding is consistent with other recently published studies, which have indicated similar levels of acceptance for such restrictive strategies aimed at safeguarding online environments. In 2021, the eSafety Commissioner surveyed 1200 Australian adults, with 78% of respondents supporting the use of age verification mechanisms as a means to verify minimum user age on pornographic websites [32]. In a similar survey conducted the following year, just 4% of young Australians believed that online pornography should remain unrestricted by age, with 59% also supporting the implementation of age verification technologies [33]. Comparable research conducted in both the United Kingdom and New Zealand has found similar levels of acceptance. Close to 83% of surveyed UK parents supported the introduction of age verification processes for access to pornographic websites [34], while 71% of young adults in New Zealand were in favor of restricting access to online pornography for teenagers and young children [35]. Our findings, therefore, add to a growing body of evidence suggesting sustained acceptance among the broader population for the use of such technologies within this context.

As a stand-alone strategy, however, these restrictive technologies have clear limitations, particularly in their ability to prevent young people from intentionally viewing explicit online content. In a survey of American high schoolers for instance, 98% of young participants stated that they had no difficulties in circumventing traditional internet filters in order to gain access to pornography [36]. Similarly, research from Australia, the United States, and the United Kingdom has consistently found a strong perception of inefficacy toward these restrictive filtering technologies among the general public [7]. It is unsurprising, therefore, that participants in this study echoed this perspective when discussing the merits of the two proposed technologies. While participants were generally supportive of the new technological approaches, few were convinced of their overall ability to prevent intentional exposure to explicit content. Both cohorts remained firm in their belief that young people were still likely to circumvent both the “SafeAgeID” and “PornScreenPro” apps. This skepticism may be attributed, in part, to the heavy reliance of both proposed technological concepts on traditional restrictive internet filtering practices to prevent exposure to online material. While novel features, such as facial imaging, may allow for a more streamlined and acceptable age verification process, both concepts remain similarly limited in their capacity to prevent intentional access due to the numerous perceived workarounds young people may exploit.

In isolation, restrictive strategies, such as age verification technologies, are therefore unlikely to provide sufficient online protection and support to young people, particularly as their sexual interests and preferences evolve. Instead, participants expressed a strong preference for informed and age-appropriate conversations as an additional and primary method for minimizing potential harm from online material. Yet crucially, participants also had a perceived deficit in their own knowledge and confidence when it came to initiating these conversations with their child, expressing a desire for additional resources to support them with this process. While comparable studies [9,35] have observed similar views from parents and caregivers toward the necessity of these conversations, there remains a dearth of literature exploring what “age-appropriate” exactly entails including the age and contexts in which these conversations should be undertaken. Acknowledging and supporting the essential role of parents and caregivers in guiding young people’s exposure to pornography is crucial, given that active parental mediation in online activities has been shown to diminish risky online behaviors and promote healthier sexual development [37-39]. Additional research is therefore required to enhance our understanding of the specific information parents require to facilitate these crucial conversations, along with determining the optimal timing and contexts for conducting these discussions with young people.

Finally, the findings of our co-design study have implications for future prototype design and development. Participants expressed a desire for more configurable options within the proposed apps to better suit their family’s needs and sociocultural understandings of pornographic content, including what types of media are screened, and to allow for families to select what they are comfortable with their children accessing. Future prototypes should also have the capability to adapt to different age groups, providing parents the ability to enforce stricter controls for younger users and gradually allow more autonomy as children mature, or as parents decide they are ready to access different content. The controls of future apps should ensure adults can watch pornographic content without being restricted or tracked unless voluntarily selected. The apps should be inclusive and accessible for all users, including those who do not speak, read, or write English, or people with disabilities. This could involve text-to-speech features, multilingual capabilities, easy-to-read fonts, and other accessibility features. Finally, supportive educational resources should be developed and provided in tandem with future technological applications to ensure better user acceptability, with a particular need for further information on user privacy and security, and broader information about the app’s design in easy-to-understand language.

### Study Limitations

The findings of this study need to be considered in light of several limitations. Participant recruitment was undertaken via an external research recruitment agency, drawing from a network of individuals who had previously indicated a willingness to participate in research. This may have introduced a level of self-selection bias, with the views expressed by our participants potentially differing from those of disadvantaged parents and young people more broadly. Workshops were also carried out within a group setting, leaving the potential for social biases to influence participant responses (eg, social desirability). This bias may have been reduced somewhat by having participants individually complete written story stems, before discussing their thoughts with the group. We were able to analyze these written data in addition to what participants were willing to say in the group discussions. The use of story-stem methodology also brings additional methodological limitations, as story completion frequently replicates the conventional structure of Western storytelling [40]. This is particularly important to note when evaluating the narratives expressed within a culturally and linguistically diverse participant cohort, whereby cultural intricacies, alternative viewpoints, and diverse narrative structures may have been overlooked or misunderstood. Finally, while the novel technological concepts proposed to participants were developed through an extensive evidence review process, they remained speculative and hypothetical prototypes, and thus, participant opinions and feedback were formed based on incomplete information and may change with supplementary details (ie, participant concerns over app security and privacy).

### Implications for Future Research

This study provides insights into the wants, needs, and concerns of parents and young people toward emerging restrictive age verification technologies. Further co-design and prototype development are needed to incorporate the findings from this study, with further iterative testing and consultation with key stakeholders to ensure user acceptability and user comfortability. Given the rapidly changing nature of the technological landscape, advances in age verification technology should be monitored closely for future relevance and application, particularly advancements in generative artificial intelligence-driven biometric systems such as facial image processing. Additionally, there is a need for the development of supportive resources for parents and other users of these proposed technologies to address concerns of privacy and user configurability. Further research is needed to better understand the information needs of parents when conducting age-appropriate conversations, including what “age-appropriate” means for different family units, and guidance on when and how to facilitate these discussions. Consideration must also be given to the sociocultural context of users, with further research needed to understand how differences in language, culture, and religion may influence parental information needs and the overall acceptability of the proposed technologies.

### Conclusions

As policy makers continue to pursue age verification and other restrictive strategies to limit children’s and adolescents’ exposure to pornography, it is vital to understand how these tools are perceived by potential users. Parents and adolescents in this study agreed that such technology is useful as a “first line of defense” against the accidental exposure of young people to online explicit materials, but that these tools are less likely to be useful or acceptable for preventing intentional pornography access. Concerns over privacy, efficacy, and adaptability need to be considered when implementing future tools or policies. Most importantly, both parents and adolescents insisted that technology alone was not the answer; parent-child conversations and comprehensive sexual health and pornography education were the ultimate solutions to addressing the potentially harmful impact of pornography on young people.

## Abbreviations

REDCap: Research Electronic Data Capture

## Data Availability

The datasets generated or analyzed during this study are not publicly available because it may be identifiable, but selected data can be made available from the corresponding author upon reasonable request.
